# GPs’ perceptions of their relationship with the pharmaceutical industry: a qualitative study

**DOI:** 10.3399/BJGPO.2021.0057

**Published:** 2021-09-29

**Authors:** James Larkin, Ivana Pericin, Maurice Collins, Susan M Smith, David Byrne, Frank Moriarty

**Affiliations:** 1 Department of General Practice, RCSI University of Medicine and Health Sciences, Dublin, Ireland; 2 School of Social Work and Social Policy, Trinity College Dublin, Dublin, Ireland; 3 School of Pharmacy and Biomolecular Sciences, RCSI University of Medicine and Health Sciences, Dublin, Ireland

**Keywords:** drug industry, general practice, Ireland, perception, qualitative research

## Abstract

**Background:**

The pharmaceutical industry invests heavily in promoting medications to physicians. This promotion may influence physicians’ prescribing behaviour and lead to inappropriately increased prescribing rates.

**Aim:**

To understand GPs’ experience of interacting with the pharmaceutical industry, and explore their views and perceptions of the impact of this interaction in general practice in Ireland.

**Design & setting:**

A qualitative design was used, and GPs practicing in Ireland were eligible.

**Method:**

A combination of purposive and snowball sampling techniques was applied and semi-structured interviews were conducted. Thematic analysis was used to develop themes from the data.

**Results:**

Twenty-one GPs and one GP trainee participated. Five themes were developed: 1) GP and pharmaceutical industry interface; 2) the industry’s methods of influence; 3) the uncomfortable relationship between GPs and industry; 4) GPs’ perceptions of being unconsciously influenced; and 5) GPs’ lack of knowledge of relevant regulations.

Participants interacted with pharmaceutical representatives in their surgery and through continuing professional development (CPD). Reported methods of influence included biased information and the offer of gifts. Most participants felt their prescribing was unconsciously influenced. A minority felt that they were only influenced in a way that improved their prescribing.

**Conclusion:**

The study shows that there can be a lack of clarity among GPs about relevant regulations and about the potential impact on prescribing of interactions with the pharmaceutical industry. Education of trainees and GPs has the potential to address this. Restrictions on interactions with the pharmaceutical industry may also play a role, although alternative CPD funding sources would need to be established.

## How this fits in

Previous research shows that physician interactions with the pharmaceutical industry can influence prescribing behaviour, and lead to inappropriately increased prescribing rates and lower prescribing quality. This qualitative study of GPs shows that the pharmaceutical industry is interacting with and influencing GPs in numerous ways. Understanding these interactions and GPs’ perceptions of them can inform strategies to ensure an appropriate relationship between GPs and the pharmaceutical industry.

## Introduction

Pharmaceutical companies spend large amounts of money on promotion of medications, with most activities directed at physicians.^1,2^ Physicians’ prescribing decisions are directly influenced by the promotion of medication.^3–6^ However, information communicated by pharmaceutical representatives is often inaccurate,^7–9^ and physicians often underestimate the impact of these promotional activities on their prescribing behaviours.^6^ A 2017 systematic review reported a *‘* *consistent association* *’* between physician interactions with the pharmaceutical industry and *‘* *inappropriately increased prescribing rates, lower prescribing quality, and/or increased prescription costs* *’* *.* ^3^ Inappropriate prescribing has the potential to harm patients, increase costs, and impair public trust in health care.

GPs are an important promotional target for the pharmaceutical industry because they initiate a high proportion of patient prescriptions.^10,11^ US evidence showed that 15% of physicians in receipt of pharmaceutical industry payments were family physicians.^12^ Previous studies have examined GPs’ interactions with the pharmaceutical industry in Ireland and found that pharmaceutical representatives were one of GPs’ primary sources for information on new drugs.^13,14^ However, these studies predate the 2007 regulation on the promotion of medicinal products,^15^ as well as the Medical Council guidance and updated Irish Pharmaceutical Healthcare Association (IPHA) Code of Practice,^16^ all described below. A 2016 meta-synthesis on interactions between pharmaceutical representatives and physicians found few studies conducted with GPs.^17^ Moreover, the majority of studies included in this meta-synthesis were conducted >10 years ago.^17^ A recent qualitative study conducted across three countries found that primary care physicians considered pharmaceutical representatives to be biased, but believed that they themselves were immune from representatives' influence.^18^

Interactions between the pharmaceutical industry and physicians are an area of public interest.^19,20^ In Ireland, these interactions are regulated by the Medical Council and a 2007 regulation.^15,21^ The Medical Council guidance is detailed, but most notably states that physicians should not accept gifts or hospitality.^21^ The 2007 regulation^15^ includes requirements for accuracy of advertising, and considers pharmaceutical representative visits within the definition of advertising. The regulation^15^ also states that free samples should be provided to prescribers on an exceptional basis only, that gifts should be inexpensive and relevant to the practice of medicine, and that reasonable hospitality is only allowed at professional or scientific events. UK regulations make similar provisions.^22^ The pharmaceutical industry has a code of practice for interacting with healthcare professionals. It was developed by the IPHA,^16^ which represents the international biopharmaceutical industry, and is based on the 2019 European Federation of Pharmaceutical Industries and Associations Code of Practice.^23^

Overall, the pharmaceutical industry is dynamic in its response to the regulatory environment,^24^ creating a need for up-to-date research. Also, Ireland offers an interesting case, as the regulatory environment, which has changed in recent years, is similar to many European countries. This research, therefore, aims to understand the experience of GPs in Ireland when interacting with the pharmaceutical industry, and to explore their views and perceptions of the pharmaceutical industry’s involvement with general practice and potential impact on prescribing.

## Method

### Study design and setting

This qualitative study is reported according to the COREQ guidelines.^25^ A phenomenological approach was applied to gather rich, in-depth data and gain a better understanding of GPs’ perceptions and everyday experiences. Semi-structured interviews were employed to facilitate the collection of in-depth information. The interview topic guide (see Supplementary Appendix A) was developed by two GPs (SS, DB), a pharmacist (FM), and two social scientists (JL, IP). The topic guide was not piloted. The main topics were:

- ‘Exploring contact’;
- ‘Meetings and information’;
- ‘Other interactions’;
- ‘Regulation’; and
- ‘Prescribing decisions’.

The interviews took place between July and September 2020.

### Participants

A combination of purposive and snowball sampling was used to recruit GPs. To increase generalisability and minimise bias, the researchers selected GPs from a mix of geographic areas, with a diverse length of time practicing, and who were of both male and female sex. Participants were recruited through the authors’ professional contacts, or suggestions from participants. In instances where there was a pre-existing relationship between researcher and participant, a researcher with no relationship to the participant conducted the interview. Invitations to participate were sent by email. Participants were provided with an information sheet and consent form before participation. Four people did not reply to requests for participation.

### Data collection

Semi-structured interviews were conducted via either telephone or the Zoom platform. Telephone interviews are useful in mitigating the complexity in accessing health professionals,^26,27^ and are considered appropriate for semi-structured interviews.^28^ Furthermore, the Zoom platform was found to be cost-effective and satisfactory by researchers and participants.^29^ Interviews were conducted by one of three researchers (JL, IP, MC), and lasted 15–45 minutes. Field notes were made after interviews and discussed among interviewers. Interviews were audio-recorded and transcribed verbatim. Transcripts were pseudonymised by assigning them a unique ID, and were returned to participants to provide an opportunity for corrections. They were then anonymised.

### Data analysis

Thematic analysis, following a six-step process,^30^ was employed to identify patterned meanings and common themes within and across the dataset. Firstly, the transcripts were read and re-read, to achieve data familiarisation, followed by line-by-line coding, where meaningful parts of texts were organised into codes and categories. The process was carried out by a single author (JL) who by employing an inductive approach, reviewed the codes to assess the commonality and differences between the interviews. Using the comparative analysis between and within transcripts, the author (JL) labelled codes based on the meaning and relationships between them into categories, which were synthesised into the main themes. To increase the confirmability of the findings, a second author (IP) read all transcripts and crosschecked the coding structure and themes developed. This individual approach, as opposed to two independent coders, was taken to facilitate the authors’ reflexivity; the sense-checker could challenge the assumptions of the primary coder.^31^ Two authors (JL, IP) discussed the categories, clarified meanings, and identified preliminary themes. Discrepancies were addressed through discussion. Analysis was conducted using NVivo (version 12) software.

### Reflexivity

Two authors (SS, DB) are GPs with a health services research background, one (FM) is a pharmacist with a health services research background, one (MC) is a medical student with a social science background, and two authors (JL, IP) have a background in social science and health services research. Three (DB, FM, SS) authors have had interactions with the pharmaceutical industry but do not currently meet with its representatives or attend events organised by it. Two authors (SS, DB) attend national educational events, which receive unrestricted funding from the pharmaceutical industry, although the pharmaceutical industry has no role in programme selection or delivery. The pharmaceutical industry has stands with representatives at the events but SS and DB avoid engaging in discussion with representatives. Authors conducting data analysis (JL, IP) reflected on and discussed their beliefs around the subject before and during analysis.

## Results

Twenty-one GPs and one GP trainee participated. Table 1 provides details of the sample. Participants’ sex and practice location closely matched national figures.^32^

**Table 1.** Participant characteristics (*n* = 22)

Participant characteristics	*n (%)*
Sex	
Male	12 (55)
Female	10 (45)
Practice role	
Salaried	10 (45)
Partner	10 (45)
Locum	1 (5)
Trainee	1 (5)
Years qualified as GP	
Trainee	1 (5)
0–4	5 (23)
5–9	3 (14)
10–14	4 (18)
15–19	2 (9)
≥20	7 (32)
Location of practice	
Urban	11 (50)
Rural	4 (18)
Mixed	7 (32)

Five themes were developed based on the data:

- GP and industry interface;
- methods of influence;
- an 'uncomfortable' relationship;
- perceptions of unconscious influence; and
- GPs’ lack of knowledge of regulations.

**Figure 1.**
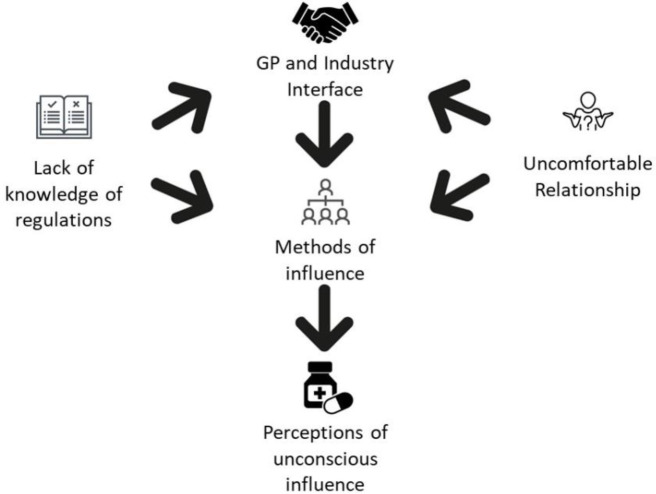
Relationships between themes.

Figure 1 illustrates the relationships between themes.

### GP and industry interface

Participants gave detailed accounts of their interactions with the industry. There were two primary settings for meetings: the general practice surgery and CPD events. Selected illustrative quotes are provided in Table 2.

**Table 2.** Illustrative quotes of the interface between GPs and the pharmaceutical industry

Topic	Quote
Meetings in practice	*‘If it was something a nurse would be dealing with, for example an intrauterine device or contraception then yes. Medical students are always fair game. Receptionist, probably not, but they might give them post-its or something.’* (P10)
	*‘Until about 10 years ago, you might end up with about two or three in a day which became a bit unmanageable so we reduced to one a day. So, for 5 days a week we all see a rep*.’ (P13)
Continuing professional development	*‘At CME or maybe at the Christmas CME like if there was a meal, they’d come in and they’d literally give, this is our product talk for 5 minutes and then off they go.’* (P2)
	*‘Conferences that I would go to there are undoubtedly* [pharmaceutical industry] *stands and tables set up for the coffee breaks, for the lunch breaks’* (P11)

CME = Continuing Medical Education. P = participant.

#### Meetings in practice

The majority of participants said that they meet pharmaceutical representatives in their surgery, either one-on-one or in groups with several GPs attending:

*‘They ask to meet with everyone, but usually they are put in a room and people see them as they have time. So sometimes it’s one-to-one and sometimes it’s in a group.’* (Participant [P]15)

Practice meetings with pharmaceutical representatives would last 5–15 minutes and varied in frequency from daily meetings to once every 3 months. In some practices, the practice nurses and/or medical students would attend.

Occasionally, pharmaceutical representatives would organise a consultant doctor to speak at a practice meeting. Seven participants chose not to meet pharmaceutical representatives in their surgery.

Two GPs believed the pharmaceutical representatives they were meeting were profiling the practice’s prescribing behaviour. They believed that pharmaceutical representatives asked *‘local pharmacists which doctor prescribes their drug’* (P20). One thought that pharmaceutical representatives were obtaining prescribing data from the company running their practice software.

#### Continuing professional development

GPs also met pharmaceutical representatives outside the practice, with some saying that they come across the pharmaceutical industry ‘*everywhere’* (P7). They described the pharmaceutical industry as ‘*intrinsically linked to CPD’* (P10) because it is very active in sponsoring and organising CPD events for GPs. The majority reported that they would encounter the pharmaceutical industry at a range of sponsored events including continuing medical education (CME) small-group meetings, conferences (for example, primary care surgical society, Irish College of General Practitioners conference, GP-trainee conference), study days, and workshops. One participant mentioned that a government minister had attended an event sponsored by the pharmaceutical industry. When sponsoring smaller events such as CME small-group meetings, a pharmaceutical representative would speak to attendees individually and/or give a brief talk about their drug at the start of the event. For their own events, pharmaceutical companies would hire a speaker, usually a consultant doctor or GP:

*‘The consultant is hired* […] *by the drug company to provide the masterclass.’* (P10)

Sponsorship of non-clinical workshops also took place; one participant described a sponsored stress reduction workshop and yoga class for GPs. Three participants said that some postgraduate training bodies received sponsorship from the pharmaceutical industry.

### Methods of influence

The interviews revealed a range of methods employed by the pharmaceutical industry to influence GPs. The most common methods were the provision of biased information, controlling discourse, and donation of gifts and contributions. Selected illustrative quotes are provided in Table 3.

**Table 3.** Illustrative quotes of the methods of influence used by pharmaceutical representatives

Topic	Quote
Biased information	*‘I’d listen but I’d be somewhat sceptical about the claims, obviously they’re telling you about all the good trials.’* (P12)
	*‘I try to have a filter so that I’m aware of the bias in their presentation and the materials.’* (P1)
Controlling discourse	*‘I’ll give you an arbitrary example, let’s imagine a supplement for vitamin D, they*[pharmaceutical company] *might bring a consultant to talk to GPs about the importance of vitamin D supplementation, and of course the company sponsoring the meeting is also providing a vitamin D supplement. Now it would never be so overt as the consultant saying you should prescribe that company’s vitamin D supplement but*[…] *that is obviously an attempt to sell a product*.’ (P17)
	*‘Opinion leaders are of course important but unfortunately I think a lot of opinion leaders are in the pockets of pharma. You know CME events should not be sponsored by pharmaceutical companies but they are.’ (P4*)
Gifts and contributions	*‘We are absolutely struggling as a new practice, like we do not have an AED at the moment and I had to look around and one might cost €1500 but if I saw a pharmaceutical representative, and I continued to see them, I could definitely ask them to pay for it and they would. And it is so attractive because ultimately it makes you more money as a GP, and I am losing out financially because I am not doing that.’* (P7)
	*‘In my experience it’s been pens, yeah cakes and sandwiches the odd time, the majority of the time it’s pens.’* (P3)

AED = automated external defibrillator. CME = Continuing Medical Education. P = participant.

#### ‘Biased’ information

Ten participants said they felt that pharmaceutical representatives were biased in their provision of information. Seven more participants felt that information provided was sometimes inaccurate. GPs felt that the presentation to them is like a ‘sales pitch’, where the information favoured the interests of the pharmaceutical representatives’ company:

*‘I think it was cherry-picked, I think what they told you was probably what painted their product in the best light.’* (P3)

Some interviewees indicated that the benefits of the product might be amplified, and they were ‘*reluctant to trust all of the information without questioning’* (P12). Participants also reported that pharmaceutical representatives would mention that a local consultant or another GP was prescribing the drug they were promoting.

A minority of participants who discussed this issue felt that the information provided by the pharmaceutical representatives they met was accurate and comprehensive.

#### Controlling discourse

At events run by the pharmaceutical industry, the talk (delivered by a consultant doctor or GP) would often be on a topic related to the medicine or device the pharmaceutical industry was presenting at the event. For events run by GP groups, the pharmaceutical industry would sometimes have input on the content:

*‘Sometimes at less mature CME groups I suppose, they set the agenda or seek to set the agenda and they say *‘* *‘*we’d like to bring down a fresh-faced respiratory physician from a hospital*’* *’*.’* (P1)

However, most participants said their groups would prevent this from happening:

*‘We exercise complete control over content and speaker.’* (P20).

However, one participant pointed out that ‘*there is invariably going to be some sort of stand or brief comment made’* (P7). Another participant, when asked why the pharmaceutical industry provided unrestricted funding, said it was because ‘*they* [the pharmaceutical industry] *got a general association within the minds of five or six key practices.’* (P20)

This effort to control discourse was also exercised when sponsoring CPD events, where the pharmaceutical industry would often organise stands with representatives from different companies. The interviewees alluded to feeling obligated and pressured to talk with pharmaceutical representatives, since ‘*they’ve sponsored the meeting and it wouldn’t happen if they weren’t there*’ (P3).

#### Gifts and contributions

Many participants said that pharmaceutical representatives sometimes bring food (for example, sandwiches or cakes), model devices, free samples, electronic devices (for example, phone charger cables), and/or stationery to practice meetings:

*‘He would end up bringing lunch for all the practice the practice manager and the secretaries and everyone.’* (P15)

Some said many of these offerings were a thing of the past, while others disclosed that it was still happening. At CPD events, participants reported that these gifts would be offered at stands. In addition, GPs said that stands at some conferences would be raffling a dinner voucher, hamper, or *‘an iPad, for those who signed their name at the stall.*’ (P14).

One participant said that they had their attendance paid for at a conference along with their food and stay at a hotel. They then described feeling pressured to prescribe the pharmaceutical representative’s drug:

*‘I didn’t need this drug. And I wasn’t gonna start changing people over to it, but this rep who was quite pushy clearly had the expectation that I was going to transfer patients.’* (P1)

GPs also mentioned practices receiving funding from the pharmaceutical industry for medical equipment, research, or a first aid course.

### ‘Uncomfortable’ relationship

#### ‘Uncomfortable’ about meeting pharmaceutical representatives

Most participants were uncomfortable about meeting pharmaceutical representatives in their practice. Participants adopted different approaches to managing this discomfort: nine reluctantly met pharmaceutical representatives (reluctant-meeters) and seven decided not to meet them (anti-meeters). A minority of six GPs were positive about meeting pharmaceutical representatives (eager-meeters). Selected illustrative quotes are provided in Table 4.

**Table 4.** Illustrative quotes on why GPs’ attitudes towards meetings with pharmaceutical representatives

Topic	Quote
Reluctant-meeters	*‘I was never madly comfortable about it but then again on a personal level I often felt sorry for the individual people. So I would out of politeness meet people.’* (P19)
Anti-meeters	‘*Meeting a rep one-to-one, who’s just there to sell a product, and won’t give me balanced views ... I have no time for that.’* (P11)
Eager-meeters	*‘The relationship I would have had with the reps over the years would have been very valuable.’ (P13*)

#### Reluctant-meeters

Reluctant-meeters were GPs who met pharmaceutical representatives but would prefer not to and felt ‘*uncomfortable*’ about meeting them. Reluctant-meeters met pharmaceutical representatives because it was practice policy, because ‘*it’s always been done*’ (P18), or because they did not want to be different:

*‘On a personal level, I always felt very uncomfortable seeing medical reps, the reason I probably saw them was because I didn’t want to be different or do something differently to the other GPs* *.* *’* (P12)

The perception of pharmaceutical representatives as being ‘pushy’ or ‘salespeople’ were common reasons mentioned for this discomfort and led some participants to reduce the number of meetings they had with pharmaceutical representatives. Many participants stressed that one reason for meeting pharmaceutical representatives was sympathy or courtesy, or to help them ‘*keep their jobs’* (P1). Others felt they were obliged to meet them because they funded medical education.

However, the most common reason for reducing meetings was time. Participants discussed having busy workloads and that pharmaceutical representatives were a low priority.

#### Anti-meeters

The primary reason that anti-meeters did not meet pharmaceutical representatives was that they did not trust their information and considered meeting pharmaceutical representatives a ‘marketing exercise’. This led some to the view that it would have a negative impact on patients through over-medicalisation or by creating a conflict of interest:

‘[I don’t meet with them] *because I do not believe that they have the patient’s interests at as their primary motivator, their primary motivating force is obviously drug profits, and the two are incompatible.’* (P4)

#### Eager-meeters

Eager-meeters felt they could learn from pharmaceutical representatives, and receive tailored information:

*‘I think it’s a good platform and forum to become aware of new medications, new advances, new regulations, new guidelines, etc.’* (P6)

For some, pharmaceutical representatives were their only source of information on new drugs. Four participants mentioned how they enjoyed the interaction, and that pharmaceutical representatives built a relationship with them.

#### ‘Uncomfortable’ about CPD funding

The majority of participants thought that it would be preferable if the pharmaceutical industry did not fund CPD as it led to bias. However, they also believed that funding was unlikely to come from elsewhere:

*‘Things that I would attend yes absolutely are sponsored* [by the pharmaceutical industry] *and it’s a really tricky one because I don’t know how you could hold a medical education conference of any kind without it.’* (P19)

Some described GP training schemes and CME groups who had made conscious decisions not to accept pharmaceutical industry funding. One reason for this decision was that participants felt there should not be a ‘vested interest’ involved in GPs’ education.

### Perceptions of unconscious influence

The majority of participants who met pharmaceutical representatives felt that they were likely to be unconsciously influenced by them (see Table 5). This was often a reluctant reflection. Sometimes it was based on the view that the pharmaceutical industry would not invest as much money as they do in promotion if it did not influence GPs. Others came to this conclusion after reading research that outlined the impact.

**Table 5.** Illustrative quotes on influencing prescribing

Topic	Quote
Unconscious influence	*‘I would probably say that I don’t feel very influenced, but I think that is not correct. I think absolutely, you can be unconsciously influenced. There is a reason why pharma reps want to see doctors and want to talk to doctors because clearly, it is an effective way of getting people to prescribe whatever drug they are promoting*.’ (P21)
Influence based on investment	*‘If they’re putting so much money into this then they must be getting something out of it.’* (P19)

Some participants felt that pharmaceutical representatives only influenced them in a way that improved their prescribing. A minority felt that they did not influence them at all:

*‘If it happens to be sponsored by a drug rep who is offering me a post-it pad, and I need a post-it pad I’ll take it, but it certainly does not skew how I would prescribe.’* (P10)

Though many of these participants felt that pharmaceutical representatives did influence other GPs.

### Lack of knowledge of regulations

Most participants lacked knowledge on regulations governing and relating to physicians’ interactions with the pharmaceutical industry. Instead, many described a general sense that things had gotten stricter:

*‘I'm not sure what the regulations are, all I know is it seems things have definitely tightened up over the years.’* (P10)

Several participants had partial knowledge of gift regulations. They mentioned that gifts were not allowed, referencing practices of the past such as trips away or expensive hotel stays. The majority of participants felt that it was appropriate to regulate this type of interaction:

*‘Probably it should be more regulated … obviously these people are trying to sell the drug that their company makes* *.* *’* (P15)

## Discussion

### Summary

GPs described meeting pharmaceutical representatives in several forums, including in their surgery and at CPD events (sponsored or organised by the pharmaceutical industry). During these meetings, participants reported that pharmaceutical representatives would use several methods to influence them, providing gifts (for example, stationery or food) and information that was perceived to be biased. Most participants felt uncomfortable about meeting pharmaceutical representatives. Many GPs reported feeling uncomfortable about the pharmaceutical industry funding CPD, with some expressing concerns that alternative funding sources may not be available. Many participants felt that their prescribing behaviour was unconsciously influenced by pharmaceutical representatives. However, others did not believe this to be the case and felt that they were only influenced in a way that improved their prescribing. Finally, GPs seemed to lack a detailed knowledge of relevant regulations.

### Strengths and limitations

The use of semi-structured interviews was flexible and allowed exploration of a range of areas, with the telephone and virtual platforms facilitating national representation. A further strength was the research team’s broad disciplinary background.

Limitations include social desirability bias,^33^ particularly for questions about conflict of interest. Also, despite the purposive sampling approach, the use of professional contacts and supplementary snowball sampling may have resulted in a biased sample, though the participants’ characteristics suggest this was not an issue. For data analysis, two independent coders would have increased reliability; however, the approach used facilitated reflexivity. Finally, data were collected in 2020, when GPs were not attending in-person CPD events or meeting pharmaceutical representatives owing to the COVID-19 pandemic, though they were asked to reflect on their normal practice.

### Comparison with existing literature

Some responders thought that interacting with pharmaceutical representatives would improve their prescribing or not affect their prescribing ‘negatively’, while also thinking that their colleagues’ prescribing would be affected. This is despite research consistently demonstrating that interactions with the pharmaceutical industry can lead to inappropriately increased prescribing rates and lower prescribing quality.^3^ However, these findings align with evidence that many physicians believe pharmaceutical representatives do not influence their own prescribing, but do influence others’ prescribing.^34–36^

Similar doubts emerged among those who accepted funding for events. They considered the funding unrestricted, implying that the funder has no influence. However, research has shown that this funding may create a feeling of reciprocity among recipients towards the pharmaceutical industry.^4^ Or, as one participant pointed out, even when funding is ostensibly unrestricted, *‘* *there is invariably going to be some sort of stand or brief comment made* *’* (P7).

Many of this study’s findings aligned with the meta-synthesis examining interactions between physicians and pharmaceutical representatives.^17^ One of the areas which was not evident in the current research was the provision of costly gifts irrelevant to patient care, such as jewellery or televisions;^17^ this may be related to regulations in Ireland that now prohibit such gifts.^15^ A notable finding was pharmaceutical representatives telling GPs that local consultants or GPs were prescribing their company’s product as a way of influencing the GPs. This has been previously documented and described as the principles of authority and social validation.^37^

Evidence was found of key opinion leaders (KOLs) acting as speakers at events organised by the pharmaceutical industry or CME groups. KOLs are senior physicians co-opted by the pharmaceutical industry to form a central part of their efforts to influence physicians.^38,39^ Recent research has shown that KOLs have a large influence on prescribing.^40^

Another important finding was that many participants perceived information from pharmaceutical representatives as biased, a phenomenon documented in other countries,^18^ despite the IPHA code stating that promotion *‘* *must be accurate, balanced, fair, objective and must not mislead either directly or by implication* *’*.^16^ The IPHA also states that claims about medicines should be based on *‘* *an evaluation of all the evidence* *’*.^16^ Despite this, participants reported a clear impression that pharmaceutical representatives provided *‘* *cherry* *-* *picked* *’* information; for example, only discussing the *‘* *good trials* *’*. These findings are in line with previous research.^7–9^ Another important finding was that participants discussed receiving hospitality and inexpensive gifts related to practice of medicine, despite Medical Council guidance stating that doctors should not receive gifts or hospitality.^21^

### Implications for practice

This research found little awareness among participants of regulations and guidelines pertaining to their relationship with the pharmaceutical industry. Educational bodies should consider providing more education on regulations, guidelines, and best practice surrounding interactions with the pharmaceutical industry. Education is particularly pertinent given the belief among some that interactions with the industry either improve their prescribing or do not influence their prescribing. Education and restricting interactions between the pharmaceutical industry and trainee physicians and medical students have been shown to reduce interactions when they begin independent practice.^41–44^

Restricting meetings with pharmaceutical representatives, along with restricting the provision of free samples and promotional materials, has the potential to reduce the impact of pharmaceutical representatives on prescribing.^45^ As evidenced by this research, some CME groups and GP practices have a policy of not interacting with the pharmaceutical industry. These policies could be introduced at the individual, practice, representative body, or regulator level. These restrictions would be beneficial for ‘reluctant-meeters’, as many want to stop interactions with pharmaceutical representatives, but face barriers such as feeling that they would be perceived as *‘* *different* *’* if they stopped meeting them. In France, certain physicians cannot receive free samples or gifts (including food).^46^ In Sweden, pharmaceutical companies cannot make financial contributions towards conference attendance.^47^ Several physician organisations have ceased accepting funding from pharmaceutical companies,^48,49^ including The College of Psychiatrists of Ireland.^50^ Healthcare bodies and regulators could adopt similar approaches, matched with structures that support greater transparency, monitoring, and enforcement. However, physicians have reacted negatively to the idea of interactions with pharmaceutical representatives being regulated.^18^ This is a pertinent issue for ‘eager-meeters’, who must be considered in the context of any restrictions.

Regulation is necessary because this research suggests that there is a discrepancy between the standards in voluntary codes for the pharmaceutical industry and the industry’s conduct.^51^ The current industry code in Ireland^16^ has no proactive means of monitoring compliance and makes no provision for sanctions. The IPHA Code Council rely on ‘complaints’ and industry ‘referrals’, but since 2013 only 13 referrals or complaints have been investigated, of which 11 were upheld. Additionally, 84% of the IPHA Code Council is comprised of members of the pharmaceutical industry (Irish Pharmaceutical Healthcare Association, *Findings of the Code Council and Appeals Board*, personal communication, 2020). Finally, this code does not apply to pharmaceutical companies who are not members of the IPHA.

Restrictions may create a funding gap. This study's findings support the call made by Alves *e* *t al^7^* for *‘* *publicly-financed, independent/non-commercial information to be integrated into health service provision.’* Previous research has shown that physicians support this.^52^ Study participants felt that there were no alternatives to pharmaceutical industry funding of CPD. Alternative sources of funding could include self-funding, state funding, and/or a hypothecated tax on the pharmaceutical industry.^53^

If funding were available, there are several potential vehicles for these unbiased information sources. The primary option is greater independent funding of conferences and CME small-groups. Another option — which has been trialled in academic evaluations — is educational outreach visits or academic detailing.^54^ Some argue that the harms associated with CPD funded by the pharmaceutical industry outweigh the benefits^55,56^ and should be foregone regardless of alternative funding availability.

In conclusion, this study shows that the pharmaceutical industry is interacting with GPs in multiple forums. During these interactions, GPs report that pharmaceutical representatives are using many of the methods of influence documented in the literature, including the use of KOLs and providing what is perceived to be biased information. Despite previous evidence to the contrary, some of the participants doubt that pharmaceutical representatives have any influence on their prescribing. There is also a lack of clarity among GPs about relevant regulations. Education of trainees and GPs has the potential to address this. Greater regulation and restrictions on interactions with the pharmaceutical industry may also play a role, although alternative funding sources for CPD would be needed.
